# Health Belief Model to Assess Mpox Knowledge, Attitudes, and Practices among Residents and Staff, Cook County Jail, Illinois, USA, July–August 2022

**DOI:** 10.3201/eid3013.230643

**Published:** 2024-04

**Authors:** Rashida Hassan, Ashley A. Meehan, Sarah Hughes, Amy Beeson, Hillary Spencer, Jourdan Howard, Lauren Tietje, Morgan Richardson, Anne Schultz, Chad Zawitz, Isaac Ghinai, Liesl M. Hagan

**Affiliations:** Centers for Disease Control and Prevention, Atlanta, Georgia, USA (R. Hassan, A.A. Meehan, S. Hughes, A. Beeson, H. Spencer, L.M. Hagan);; Chicago Department of Public Health, Chicago, Illinois, USA (H. Spencer, J. Howard, L. Tietje, M. Richardson, A. Schultz, I. Ghinai);; Cermak Health Services of Cook County and Cook County Health, Chicago (C. Zawitz)

**Keywords:** mpox, monkeypox virus, viruses, correctional facilities, health belief model, qualitative research, Illinois, United States

## Abstract

In summer 2022, a case of mpox was confirmed in a resident at the Cook County Jail (CCJ) in Chicago, Illinois, USA. We conducted in-depth interviews with CCJ residents and staff to assess mpox knowledge, attitudes, and practices; hygiene and cleaning practices; and risk behaviors. We characterized findings by using health belief model constructs. CCJ residents and staff perceived increased mpox susceptibility but were unsure about infection severity; they were motivated to protect themselves but reported limited mpox knowledge as a barrier and desired clear communication to inform preventive actions. Residents expressed low self-efficacy to protect themselves because of contextual factors, including perceived limited access to cleaning, disinfecting, and hygiene items. Our findings suggest correctional facilities can support disease prevention by providing actionable and tailored messages; educating residents and staff about risk and vaccination options; and ensuring access to and training for hygiene, cleaning, and disinfecting supplies.

In May 2022, mpox cases were identified in several nonendemic countries, including the United States, predominately among gay, bisexual, and other men who have sex with men (*1*–*4*). During the outbreak, transmission frequently occurred from contact with mpox lesions on the skin or mucosal surfaces during sexual activity (*5*). In summer 2022, vaccination campaigns began for persons exposed to or at higher risk for mpox (*6*,*7*).

Persons living in congregate settings, such as correctional and detention facilities, are at increased risk for many infectious diseases. Monkeypox virus (MPXV) transmission has been linked to communal housing and types of activities common in correctional facilities, including sharing clothing, linens, and personal items (*8*). In addition, access to hygiene and sanitation supplies in such facilities is sometimes limited (*9*). Mpox outbreaks were identified in correctional facilities in Nigeria, but the mode of transmission was not identified (*10*,*11*). At the time of this investigation, little was known about the acceptability and feasibility of mpox vaccination in correctional facility settings.

On July 22, 2022, mpox was confirmed in a person detained in Cook County Jail (CCJ) in Chicago, Illinois, USA (*12*), the first mpox case identified in a US correctional or detention facility. The Chicago Department of Public Health (CDPH) and the Centers for Disease Control and Prevention (CDC) investigated and found no higher-risk exposures or additional cases. CDPH and CDC determined that transmission in similar settings might be limited in the absence of higher-risk exposures, such as sexual contact (*12*). We conducted interviews at CCJ to assess mpox knowledge, attitudes, and practices among residents and staff; evaluate the acceptability and feasibility of vaccination for postexposure prophylaxis for mpox among residents; and identify information to include in mpox education materials for persons living and working in similar facilities.

## Methods

### Study Participants

During August 2–4, 2022, we conducted in-depth interviews with CCJ residents and staff. Among 57 potentially exposed residents who had shared a dormitory-style housing unit with the mpox case-patient, 19 were still residing in CCJ at the time of the investigation. We invited all 19 residents to participate, in addition to a purposeful convenience sample of 13 staff member who worked in various roles at CCJ during our investigation. Staff provided verbal consent; residents provided written consent by making a nonidentifying mark on a document that included details of the interview process, voluntary nature of participation, and confidentiality protections. This investigation was part of a public health response to an ongoing outbreak. It was reviewed and approved by CDC and conducted consistent with applicable federal law and CDC policy (*13**–**17*).

### Data Collection

We developed a semistructured interview guide with questions on knowledge, attitudes, and practices regarding mpox and postexposure prophylaxis, hygiene and cleaning practices, and behaviors in jail that could lead to mpox transmission. Resident interviews were conducted in semiprivate spaces, far enough away from other residents and staff to provide audio privacy. A custody officer remained nearby, maintaining visual contact. Staff interviews were conducted in private spaces. All resident interviews were conducted by 2 interviewers, 1 leading the interview and 1 taking detailed notes. Some staff interviews were conducted by a single interviewer because of time constraints. All interviewers were trained on in-depth interview techniques, and interviews lasted ≈30–45 minutes.

### Data Analysis

We analyzed data in 2 phases. First, we developed an a priori matrix to organize and analyze findings (*18*–*20*) to make evidence-based recommendations to improve immediate mpox response activities (*12*). Columns included predetermined topics aligned with interview questions. We entered participant responses into each row and summarized responses across row and topic, enabling rapid identification of findings. Key themes were compiled by reviewing the matrix entries, interview notes and summaries, and organizing findings and common themes. The study team discussed themes to summarize and reach consensus.

We later reassessed those data using the health belief model, a framework used to understand health behaviors and develop strategies to motivate behavior change (*21*,*22*). The model includes predictors for human behavior, such as perceived susceptibility to a disease or condition, perceived severity of illness, perceived benefits to taking action, perceived barriers to action, cues to action, and self-efficacy (*21*,*22*). Organizing the data around that framework further informed health promotion efforts in CCJ and similar settings.

## Results

Of 19 eligible residents, 16 (84%) consented to participate; all 13 staff consented. Residents ranged in age from 21 to 62 (median 43) years; all identified as male and as heterosexual/straight (Table 1). Nine (56%) identified as non-Hispanic Black, 4 (25%) non-Hispanic White, 2 (13%) Hispanic/Latino, and 1 (5%) non-Hispanic Asian. Participants spent 1–7 (median 5) nights in the same housing unit as the resident with mpox. Among the 13 staff, 7 (54%) worked in healthcare, 4 (31%) in custody, and 2 in other roles (Table 2). Interview themes were organized within the health belief model constructs (Table 3).

**Table 1:** Characteristics of 16 resident qualitative interview participants in study assessing mpox knowledge, attitudes, and practices among residents and staff, Cook County Jail, Illinois, USA, July–August 2022*

Characteristics	Value
Median age, y (range)	43 (21–62)
Median no. nights potentially exposed (range)	5 (1–7)
Male sex	16 (100)
Race or ethnicity	
Black or African American, non-Hispanic	9 (56)
White or Caucasian, non-Hispanic	4 (25)
Hispanic or Latino	2 (13)
Asian, non-Hispanic	1 (6)
Sexual orientation	
Heterosexual or straight	16 (100)
Ever accepted mpox PEP†	9 (56)

*Values are no. (%) participants except as indicated. PEP, postexposure prophylaxis. †Includes residents who accepted mpox PEP initially and when reoffered. Acceptance rates were higher among persons offered PEP in individual or small group sessions compared with those offered PEP in a large group. Information on the number receiving a second dose of PEP was unavailable.

**Table 2:** Characteristics of 13 staff qualitative interview participants in study assessing mpox knowledge, attitudes, and practices among residents and staff, Cook County Jail, Illinois, USA, July–August 2022*

Staff role	No. (%)
Healthcare	7 (54)
Custody	4 (31)
Other	2 (15)

**Table 3:** Summary of findings and illustrative quotes from study assessing mpox knowledge, attitudes, and practices among residents and staff, Cook County Jail, Illinois, USA, July–August 2022*

Construct	Residents	Staff
Perceived susceptibility to mpox	Moderate to high. Residents perceived increased risk for infection due to structural factors of being in a correctional/detention setting.	Moderate to low. Staff perceived trust in the effectiveness of PPE but acknowledged increased risk due to the nature of correctional/detention settings.
	“I’m a clean freak type, constantly disinfecting and I stay away from a lot of people, but I’m not sure about things outside of my control.” (CCJ resident)	“I think it’s unlikely that I will get monkeypox, but my concern is heightened because of the environment I work in.” (CCJ staff, nurse)
Perceived severity of potential mpox illness	Uncertain. Residents and staff were not sure how severe mpox illness would or could be, or how severity might differ based on the presence of underlying conditions.	
	“I’m a diabetic…does it affect me? With COVID they said people with diabetes and older people need to be concerned…yeah, it may mess me up especially because I got diabetes.” (CCJ resident)	“I’m not sure how sick I would get. I don’t know how severe this is.” (CCJ staff, custody officer)
Perceived benefits of behavior change to prevent mpox	Some residents had previous knowledge about other vaccines and felt that receiving vaccination for mpox would protect their health. Residents also wanted to avoid bringing mpox home to their families once released from CCJ.	Staff described wanting to engage in mpox prevention behaviors to protect themselves and to avoid bringing mpox home to their families after work.
	“Is there any way to get tested [for mpox]? Cause it’s a lot of people in my cell and I just want to make sure…and I don’t want to take it back to my family.” (CCJ resident)	“We have grandkids and kids at home we don't want to take it home to.” (CCJ staff, other role)
	“The medical officers offered vaccine and I accepted. I was given no information, but I said let me get protected before anything gets out of hand…I just want to be safe.” (CCJ resident)	
Perceived barriers to mpox preventive actions	Residents described barriers to preventive actions related to lack of knowledge and information about mpox and mpox PEP. They also described rumors about mpox that could be a barrier for others. Residents also perceived limited availability and insufficient quality of cleaning supplies and personal hygiene items (especially soap), which acted as a barrier for them.	Staff described primarily knowledge and information barriers to mpox prevention. Staff also described rumors about mpox that could be a barrier for others.
	“I don’t know how [the vaccine] works or what’s in it. If I were to take it, I would have to learn more about it.” (CCJ resident)	“As long as I follow PPE protocol, I'll be ok.” (CCJ staff, nurse)
	“I was told it’s from Boystown† and it’s a homosexual disease, I’m not sure if that info is true…Other inmates are pretty upset and homophobic, saying wild stuff.” (CCJ resident)	“I’m not sure if this is real, but people say it’s largely among the homosexual community. I don't know that I agree.” (CCJ staff, custody officer)
	“The facility doesn’t keep disinfectant on the deck [dormitory]. They're supposed to bring them every day, but it’s variable.” (CCJ resident)	
Cues to action to engage in mpox preventive actions	A confirmed mpox case within CCJ served as the cue to residents and staff to engage in preventive actions. Both residents and staff expressed the need for timely, clear communication to inform these actions.	
	“If I was in charge of telling people, I would tell them flat out the truth and not leave anything out.” (CCJ resident)	“Let people know what’s going on in real time, not a day or two later. Rumors will start to spread.” (CCJ Staff, custody officer)
Self-efficacy to engage in mpox preventive actions	Residents felt limited self-efficacy to protect themselves from mpox in the jail setting due to limited mpox knowledge, perceived limited access to healthcare and cleaning and hygiene supplies, perceived insufficient communication, and facility factors like communal housing.	Healthcare staff had higher levels of self-efficacy because of their medical training, availability and knowledge of recommended PPE, and experience caring for patients with other infectious diseases. Staff in custody roles expressed more limited self-efficacy, due to a closer physical proximity to residents, limited knowledge of mpox and prevention methods, and perceived insufficient communication.
	“There’s no way to protect yourself… ‘stay 6 feet from other people’ which is hard because the bunks are not 6 feet apart from each other.” (CCJ resident)	“COVID-19 has opened our eyes and we’ve gotten used to taking care of these things as they come…The nurses here have been trained to handle this.” (CCJ staff, healthcare provider)
		“I don’t know how likely it is that I would get [mpox], every now and again I have to go hands on with [a detainee]…Whenever they leave the tier, we always have to pat them down.” (CCJ staff, custody officer)

*Categories are organized according to the health belief model construct (*21*,*22*). CCJ, Cook County Jail; PEP, postexposure prophylaxis; PPE, personal protective equipment. †Boystown, also known as Northalsted, is a historical LGBTQ+ neighborhood in Chicago, Illinois.

### Perceived Susceptibility to Mpox

Residents reported varied levels of concern about mpox, from not concerned at all to very concerned, and felt that residing in CCJ heightened their risk. Some residents reported keeping to themselves and therefore felt their risk was low. However, most residents were concerned about factors outside their control, such as communal housing, that could increase their risk. For some residents, their heightened sense of susceptibility led to more conservative behaviors, such as frequent handwashing and avoiding social interactions or recreational activities.

Similarly, although staff thought their risk for MPXV infection was low, they perceived working in a jail inherently increased their risk for contracting infectious diseases. Some staff expressed confidence in their knowledge of infection prevention and control practices, such as using personal protective equipment, but others understood those tools might not guarantee protection.

### Perceived Severity of Potential Mpox Illness

Residents and staff were unsure how severe illness would be if they contracted mpox. However, some drew connections to COVID-19 and wondered if persons with weakened immune systems or underlying conditions would have more severe illness.

### Perceived Benefits of Behavior Change to Prevent Mpox

Residents and staff described several benefits to mpox prevention behaviors, including preventing transmission to their families. Residents were concerned that quarantining or isolating because of mpox exposure or MPXV infection could delay their release from jail. Those concerns motivated residents to want to protect themselves, but they felt they did not have sufficient knowledge about prevention options. Several residents felt they did not receive adequate information about the vaccine when it was offered, including information on safety and side effects (*12*). However, some residents reported they chose to get vaccinated, relying on previous knowledge that vaccination reduces risk for other illnesses.

### Perceived Barriers to Mpox Preventive Actions

#### Limited Knowledge and Rumors about Mpox

Most residents and some nonhealthcare staff described limited knowledge about mpox symptoms, prevention, or vaccines as a barrier to preventive action. Many residents reported they first heard about mpox while detained in CCJ, after news about the mpox case in CCJ was reported to the public. Many residents did not remember being notified by staff about possible exposure or reported that the information was difficult to understand because it was provided to the entire housing unit at once. Residents wanted more information about the vaccine and other prevention options.

At the time of interviews, healthcare staff had recently completed an online mpox training, covering transmission, prevention, and vaccines, which they felt provided knowledge to protect themselves. Nonhealthcare staff had varying levels of mpox knowledge. Like residents, most staff reported their mpox-related information came from the news or others in CCJ, including information about the mpox case at CCJ; they had not received mpox training, and they felt unsure how to protect themselves.

Several residents and staff reported hearing rumors that mpox was a “gay disease.” They reported being hesitant to believe the rumors and did not describe rumors as a barrier to taking preventive action. However, residents and staff mentioned those rumors spreading within CCJ and were concerned the rumors might act as a barrier for others.

#### Challenges Accessing Healthcare and Supplies

Many residents were willing to report potential mpox symptoms to healthcare staff but felt that follow-up on requests for healthcare services in general was inconsistent. Residents felt they had inadequate access to cleaning, disinfecting, and hygiene supplies. Residents were issued bar soap at no cost, but many reported quickly running out of soap because they used it for handwashing, showering, and washing dishes and clothes. Most residents felt there was not enough soap available, especially if they were unable to purchase additional soap from the commissary. Residents believed supplies provided to clean and disinfect their living spaces were ineffective because the disinfectant was unlabeled and smelled like vinegar. Residents also described challenges accessing brooms, mops, and buckets. Staff believed the disinfectant was in line with guidance for disinfectants for viral pathogens but felt residents were unsure how to use it.

### Cues to Action to Engage in Mpox Prevention

The mpox case within CCJ was the cue to action for residents and staff to protect themselves; however, many residents and nonhealthcare staff did not feel they had the information or resources to do so. Participants desired timely, clear communication about possible mpox exposure and prevention options, which they felt they had not received. Participants felt clear communication would help quell rumors, enable persons to better protect themselves and others, and improve relationships among staff in different roles and between staff and residents.

### Self-Efficacy to Engage in Mpox Preventive Actions

Self-efficacy to engage in mpox preventive actions varied. Many residents expressed low self-efficacy because of limited mpox knowledge, perceived limited access to healthcare and cleaning and hygiene supplies, perceived insufficient communication about their risk, and facility factors such as communal living. Healthcare staff reported greater self-efficacy because of medical training, knowledge and availability of personal protective equipment, and experience caring for patients with other infectious diseases. However, staff in other roles described limited self-efficacy because of more extended physical proximity to residents, including contact that was unpredictable and outside their control, limited knowledge of mpox and prevention methods, and perceived insufficient communication about their risk.

## Discussion

Our findings highlight the perspectives of jail staff and residents on communication, infection prevention, and vaccination after an mpox case was confirmed in CCJ. The rapid data analysis enabled us to provide real-time, stakeholder-informed recommendations to enhance mpox prevention and control efforts in CCJ and to create a toolkit to make those recommendations available to other correctional and detention facilities nationally (*12*,*23*–*25*).

Staff and residents at the jail described several barriers to engaging in mpox preventive actions: limited knowledge about mpox, risk, and postexposure prophylaxis; perceived insufficient communication about the mpox case and potential exposures; perceived inadequacy of cleaning and hygiene supplies among residents; and reported limitations in healthcare access among residents. Staff and residents had varied levels of self-efficacy but shared the need for clearer and more timely communication to prevent the spread of misinformation and empower them to make informed decisions.

Because of unique contextual factors related to disease transmission in correctional and detention facilities, providing tailored education and messages for residents and staff during public health emergencies and specific guidance about preventive actions available in these settings are critical (*24*,*25*). Previous studies have described the challenges of health promotion within correctional settings, including the influence of social networks and norms on health behaviors and the need to build rapport and trust to promote behavior change (*26*–*28*). In addition, ensuring residents and staff have access to sufficient hygiene supplies and that they know what cleaning and disinfecting supplies are available, how to request them, and how to properly use them is essential. Lessons learned from our findings and from past health education efforts in correctional settings, including during the COVID-19 pandemic (*29*–*31*), can inform strategies for future public health efforts.

The first limitation of our analysis is that interviews were limited to staff and residents in CCJ at the time of our investigation, and we were unable to speak to residents who had already left CCJ or staff not working during our investigation. Another limitation is that residents were within eyesight of custody officers during interviews, and some residents might have been uncomfortable disclosing sensitive information. Finally, our analysis was limited to a small sample and 1 facility; findings might not be generalizable to other settings.

In conclusion, correctional and detention facilities can support prevention of mpox and other infectious diseases by providing exposure notification and prevention messages that are destigmatizing, actionable, and tailored to the population and setting; by educating residents and staff about their infection risk and vaccination options; and by ensuring residents have access to sufficient hygiene, cleaning, and disinfecting supplies and training on how to use them. Including rapid qualitative analyses as part of the mpox case investigation helped accomplish timely development of setting-specific disease prevention tools that were informed by the residents and staff living and working in the affected facility. Rapid qualitative approaches, together with the inclusion of behavioral scientists and communication specialists to response teams, could be valuable additions to outbreak investigations of emerging infectious diseases in correctional settings. These tools can highlight population-specific challenges and barriers and provide actionable information for correctional settings to inform tailored prevention materials during future disease responses.
